# Induction of mitochondrial dependent apoptosis and cell cycle arrest in human promyelocytic leukemia HL-60 cells by an extract from *Dorstenia psilurus*: a spice from Cameroon

**DOI:** 10.1186/1472-6882-13-223

**Published:** 2013-09-10

**Authors:** Constant Anatole Pieme, Santosh Kumar Guru, Pantaleon Ambassa, Suresh Kumar, Bathelemy Ngameni, Jeanne Yonkeu Ngogang, Shashi Bhushan, Ajit Kumar Saxena

**Affiliations:** 1 Faculty of Medicine and Biomedical Sciences, University of Yaounde I, P.O. BOX 1364, Yaounde, Cameroon; 2 Cancer Pharmacology Division, Indian Institute of Integrative Medicine, Canal Road, Jammu 180001, India; 3 Faculty of Science, University of Yaounde I, P.O. BOX 812, Yaounde, Cameroon

**Keywords:** *Dorstenia psilurus*, Spice, Apoptosis, HL-60, ROS, Mitochondrial membrane potential

## Abstract

**Background:**

The use of edible plants is an integral part of dietary behavior in the West region of Cameroon. *Dorstenia psilurus* (Moraceae) is widely used as spice and as medicinal plant for the treatment of several diseases in Cameroon. The aim of this study is to investigate the cytotoxic and apoptotic potential of methanol extract of *D. psilurus* in human promyelocytic leukemia (HL-60) cells and prostate cancer (PC-3) cells.

**Methods:**

Cytotoxicity of *D. psilurus* extract was tested in HL-60 and PC-3 cells using 3-(4, 5-dimethylthiazol-2-yl)-2,5-diphenyltetrazolium bromide (MTT) reduction assay and flow cytometric methods

**Results:**

The methanol extract of *D. psilurus* have significant *in vitro* cytotoxic activity in HL-60 cells and PC-3 cells with IC_50_ value of 12 ±1.54 μg/ml and 18 ± 0.45 μg/ml respectively after 48 h. The mechanism of antiproliferative activity showed that after 24 h, *D. psilurus* extract induces apoptosis on HL-60 cells by the generation of reactive oxygen species (ROS) along with concurrent loss of mitochondrial membrane potential, modification in the DNA distribution and enhance of G2/M phase cell cycle.

**Conclusion:**

The extract induces apoptosis of HL-60 cells associated with ROS production, loss of mitochondrial membrane potential and apoptotic DNA fragmentation.

## Background

Spices have served humans as valuable components of seasonings, medicines, and have played a significant role in maintaining human health and improving the quality of human life for thousands of years. There is no doubt that increasing the intake of spices is one of the most effective, convenient and economical ways in which we can fortify ourselves against infectious diseases and related cancers [1]. To date, hundreds of compounds have been identified as potential remedies of cancer, several of which are active ingredients in herbs and spices [2]. The use of herbal remedies based on spices as a kind of complementary and alternative medicine (CAM) is documented in the population especially among cancer patients [3-5]. The spice based herbal medicines and the constituents have been reported to inhibit the proliferation of cancer cells directly. *In vitro* studies indicate that herbs, spices, and their bioactive components can inhibit, and sometimes induce pathways that regulate cell division, cell proliferation, detoxification, in addition to the inflammatory and immune response [2,6]. For instance, ursolic acid, a bioactive component in some herbs and spices, suppressed TNF-induced expression of genes regulated by NF-κB (cyclin D1, COX-2, and MMP-9) which are involved in tumor initiation, promotion, and metastasis [7]. In Cameroon, several studies have been carried on the cytotoxic activity of some spices on different cell lines [8-10].

*Dorstenia psilurus (Moraceae)* is a plant from widely used in Cameroon, Africa and Madasgascar for different purposes. Plant drugs from this genus have shown a broader acceptability among some indigenous populations [11,12]. The roots of *D. psilurus* are used in Cameroon as spices in the traditional meal called “*Nkui*” and “*Nah poh*”. It is also claimed to preserve health, prevent ageing, to improve the wellbeing and freshness of young mothers after several pregnancies. The chemical composition in terms of macronutrients and micronutrients of roots extract of *D. psilurus* has been reported [13]. A decoction of leaves of *D. psilurus* is used in Cameroon to treat rheumatism, snake bites, headache and stomach disorders, arthralgia, cardiovascular disorders, diuretic, tonic, stimulant, analgesic, inflammatory diseases and cancers [14,15]. Phytochemical analysis of root extract of this plant demonstrated the presence of prenylated flavonoids, stearyl-p-coumarate, stearylferulate, benzofuran derivatives, Dorsilurins C, D and E [16] and Dorsilurins (F-K) [17]. The methanol extract of *D. psilurus* has been reported to have anti-inflammatory property [12]. Some biological activities of root extract of *D. psilurus* such as the scavenging property on DPPH radical [12], anti-amylase, anti-lipase and antioxidant activities [18] and hypertensive effects, glucosidase inhibitors property [17], antibacterial activity [19] and cytotoxicity activity on MiaPaCa-2 (panceatic), CCRF-CEM, CEM/ADR5000 (leukemia) cells have been demonstrated [8].

However, there are no studies on the cytotoxicity or apoptosis inducing properties of the roots extracts of *D. psilurus* on human promyelocytic leukemia (HL-60) and prostate cancer (PC-3) cell lines. Therefore this research aimed to determine the cytotoxic of the methanol extract of *D. psilurus (*root) on two cell lines (HL-60 and PC-3) and investigate its toxicological mechanism on the most sensitive cells.

## Methods

### Plant material and extraction

The roots of *Dorstenia psilurus* (*D. psilurus)* were collected at Komako in the West Region of Cameroon and identified by Mr Victor NANA, of the National Herbarium of Cameroon, in December 2010. A voucher specimen (1649/SRF/CAM) was deposited at the National Herbarium Yaounde, Cameroon. The roots of *D. psilurus (DP)* were air-dried and ground. The powdered plant material (150 g) was macerated in MeOH (1 l) for 24 h at room temperature and then repeated once. The diluted extract was concentrated under reduced pressure to afford 40 g of a dark residue.

### Cell culture

Human promyelocytic leukemia (HL-60 cells) and prostate cancer (PC-3 cells) were obtained from European Collection of Cells Culture (ECCC), Sigma Aldrich, India. They were grown in RPMI-1640 medium containing 10% Foetal bovine serum (FBS), penicillin (100 IU/ml) and streptomycin (100 μg/ml medium). The cells were culture in the incubator (Thermocom Electron Corporation, USA) at 37°C, 5% CO_2_; 98% humidity. Cells were used for different assays during logarithmic growth phase while the untreated control cultures received only the vehicle (DMSO <0.1%).

### Cells viability and treatments

The human promyelocytic leukemia (HL-60 cells) and prostate cancer (PC-3 cells) were seeded in different 96 well plates containing 15x10^3^ and 6x10^3^ cells/100 μl/well, respectively. The cultured cells were then treated the same (triplicate wells per condition) by the addition of 100 μl of serial dilutions of the DP extract dissolved in DMSO to give a final concentration of 30, 10 and 1 μg/ml. For PC-3, the extract was added after 24 h of incubation. In addition, the DMSO alone was added to another set of cells as the solvent control (DMSO <0.1%). The cells were then incubated for another 48 h prior to the addition of 20 μl of 2.5 mg/ml solution of 3-(4, 5-dimethylthiazol-2-yl)-2, 5-diphenyltetrazolium bromide (MTT) into each well. The incubation was continued for another 3 h before the media was removed. A mixture of DMSO (150 μl) was added to each well and mixed to ensure dissolving of the crystal formazan before the absorbance at 570 nm was measured. Three replications of each experiment were performed and fifty percent of inhibitory concentration (IC_50_) of each extract was calculated.

### DNA content and cell cycle phase distribution

HL-60 cells (1x10^6^ cells/2 ml/well) were treated with DP at 20, 50, 100 μg/ml for 24 h. They were harvested and washed with 1 ml of PBS, then centrifuged 400 g for 5 min at 4°C. The pellet was suspended in 100 μl of PBS and 900 μl of hypertonic buffer (PI-25 μg/ml, RNAase-40 μg/ml, sodium citrate-0.1% and Triton-100X-0.03%) and incubated at 37°C in dark for 20 min. Finally, cells were analyzed immediately on flow cytometer FACSCalibur (Becton Dickinson, USA). The data were collected in list mode on 10,000 events and illustrated in a histogram, where the number of cells (counts) is plotted against the relative fluorescence intensity of PI (FL-2; λem: 585 nm; red fluorescence). The resulting DNA distributions were analyzed by Modfit (Verity Software House Inc., Topsham, ME) for the proportions of cells in G_0_-G_1_, S- phase, and G_2_-M phases of the cell cycle [20].

### Hoechst 33258 staining of cells for nuclear morphology

HL-60 cells (2x10^6^ cells/3 ml/well) were treated with DP extract at different concentration of extract for 24 h. They were collected, centrifuged at 400 g and washed once with PBS. A solution of Hoechst (Hoechst, 10 μg/ml; citric 10 mM; Na_2_HPO_4_ 0.45 M; Tween-20 0.05%) was added in each tube and kept in the dark at room temperature for 30 min. The mixture was then washed once with PBS and the pellet resuspended in 100 μl of PBS/glycerol (1:1). The solution (10 μl) was poured into the slide and observed for nuclear morphology alterations under fluorescence microscope (Olympus X 70, magnification 20 X).

### Mitochondrial membrane potential (MMP) assay

HL-60 cells (1x10^6^ cells/2 ml/well) were treated with DP extract at different concentrations for 24 h. Thirty minutes before the end of the experiment, the cell culture was treated with Rhodamine-123 (200nM) and keep in the dark for 30 mn. Cells were then collected, centrifuged (400 g; 4°C; 5 min), the pellet was washed with 1 ml of PBS and centrifuged as mentioned earlier. The fluorescence intensity of 10,000 events was analyzed in FL-1 channel on BD FACSCalibur (Becton Dickinson, USA) flow cytometer. The decrease in fluorescence intensity because of mitochondrial membrane potential loss was analyzed in FL-1 channel and the change of in potential membrane (∆ψm) was assessed by comparing fluorescence.

### Reactive oxygen species (ROS) assay

ROS production was monitored by flow cytometry using 2’, 7’-dichlorodihydrofluorescin diacetate (DCFH_2_-DA). This dye is a stable non polar compound that readily diffuses into cells and is hydrolyzed by intracellular esterase to yield 2’,7’-dichloro dihydrofluorescin (DCFH), which is trapped within the cells. Hydrogen peroxide or low molecular weight peroxides produced by the cells oxidize DCFH to the highly fluorescent compound 2’,7’-dichlorofluorescein (DCF). Thus, the fluorescence intensity is proportional to the amount of hydrogen peroxide produced by the cells. Briefly, HL-60 cells (1x10^6^ cells/2 ml/well) were treated with DP at different concentration for 24 h. Thirty minutes before the end of the experiment, the cell culture was treated with DCFH_2_-DA (50 μM) and keep in the dark. Cells were then collected, centrifuged (200 g; 4°C; 5 min) and the pellet was washed with 1 ml of PBS and centrifuged as mentioned earlier. The pellet was suspended in 500 μl of PBS and the fluorescence was assessed by comparing two fluorescence emission 480 nm/530 nm using a flow-cytometer (BD-LSR).

### Statistical analysis

The viability experiments were done in triplicates and each data point represents the average of at least 3 independent experiments. The data was expressed as mean ± SD. In order to carry out statistical analysis, the data was analyzed using SPSS (Version 11.5; SPSS Inc.,) and M.S. Office, Excel software. One way analysis of variance technique was applied to observe the significance between the groups. The post hoc test Duncan’s multiple range test was performed to know the significant difference among the groups. Entire statistical analysis was carried out at p < 0.05.

## Results

In this study, the human promyelocytic leukemia cell line, (HL-60), was used to investigate the capability of the methanol extract of DP to induce apoptosis and elaborate its molecular mechanism. After extraction, the yield of DP extract was 26.6%.

### Effects of DP extracts on the proliferation of HL-60 and PC-3 cells

**Figure 1:**
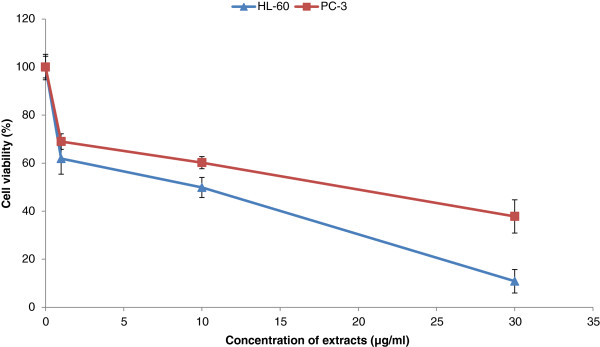
Viability of PC-3 and HL-60 cells after 48 h treatment with DP extract (n = 3).

Using a conventional tatrazolium-based colorimetric cell proliferation assay, we screened the antiproliferative activity of DP at 100 μg/ml for 48 h. The results demonstrated that these extracts reduced the cell viability between 6.25 and 6.87% depending on the cells. The viability of both cells decrease from 60.4 ± 2.84% and 65.51 ± 6.70% respectively for HL-60 and PC-3 at 1 μg/ml to 6.25 ± 2.31% and 6.87 ± 3.15% at 100 μg/ml after treatment. Further, the cytotoxicity effects of this extract were performed at different concentrations (1–100 μg/ml) as shown in the Figure 1. After 48 h, the extract of DP demonstrated a significant reduction of cell viability on both cell lines in a dose dependent manner. The fifty percent inhibitory concentrations (IC_50_) were 12 ± 1.54 μg/ml and 18 ± 0.49 μg/ml respectively with HL-60 and PC-3 (Table 1). This result confirmed that DP has significant cytotoxicity property on HL-60 cells and this cell line was chosen for the other experiments.

**Table 1:** Cells viability and fifty percent inhibition of HL-60 and PC-3 cells after 48 h

**Cell lines**	**Viability (%)**	**IC**_**50**_
	**100 μg/ml**	**(μg/ml)**
HL-60	6.25 ± 2.31	12 ± 1.5
PC-3	6.87 ± 3.15	18 ± 0.49

Results are represented as mean ± SD, n = 3.

### Morphological changes of apoptotic treated HL-60 cells with DP extract

**Figure 2:**
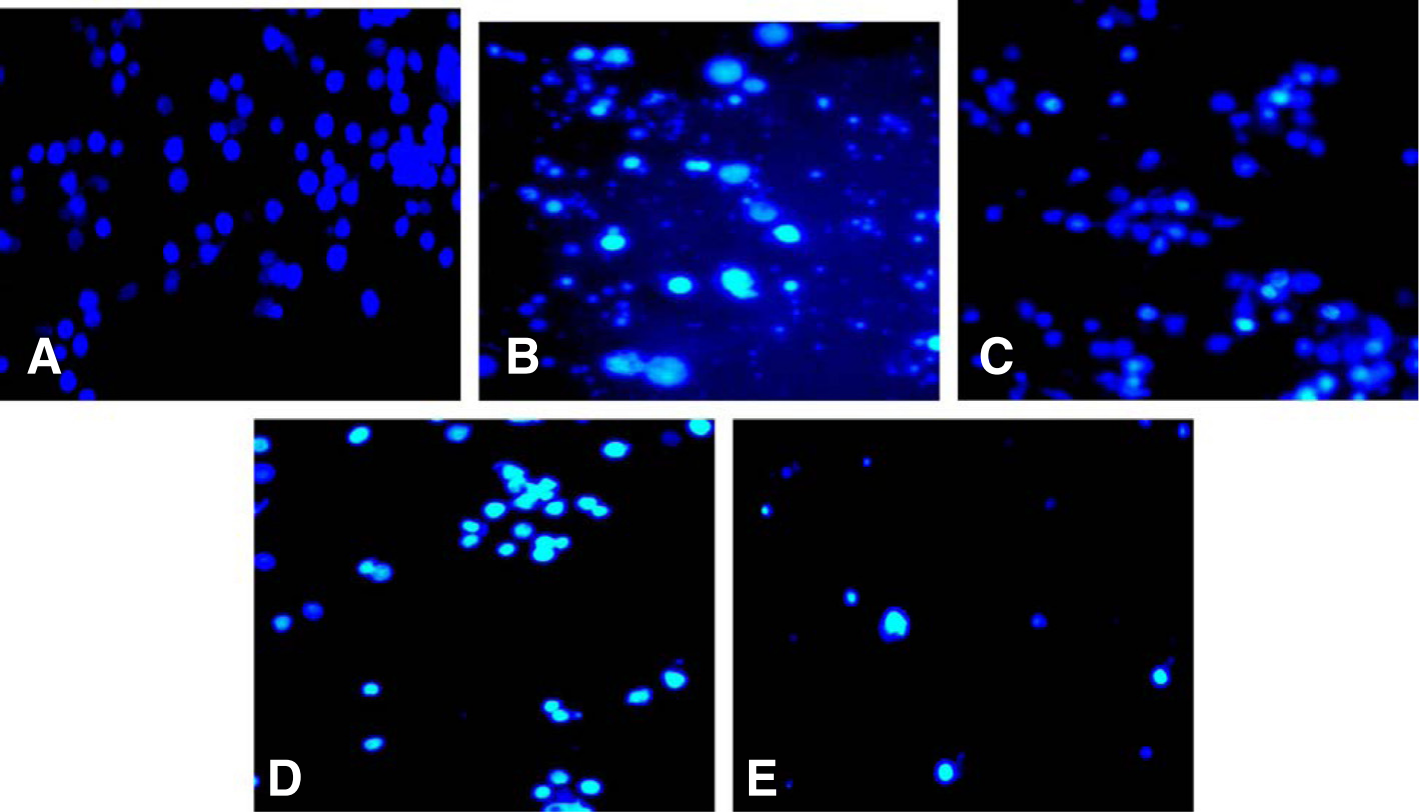
Effect of DP extract on nuclear morphological changes of HL-60 cells after 24 h and observation under fluorescence microscope (Olympus, Tokoy, Japan; magnification · 200). (A): control; (B): Camptotecin (2 μM); (C): 20 μg/ml; (D): 50 μg/ml; (E): 100 μg/ml.

Nucleosomal DNA fragmentation is the result of activation of endogenous endonuclease. To investigate whether the DP extract can induce apoptosis and nuclear modification on HL-60 cells after 24 h of treatment, the Hoechst 33258 staining was also carried out at different concentrations (20, 50 and 100 μg/ml). It is a membrane-permeable blue fluorescent dye which stained cell nucleus. The results show that the untreated HL-60 cells present the uniformly light blue nuclei under fluorescence microscope demonstrating that the cells are in healthy conditions (Figure 2A). DP-treated HL-60 cells exhibited a bright blue color (Figure 2) confirming the dead of cells. DP-treated HL-60 cells showed condensed and marked fragmented nuclei in a time-dependent manner (Figures 2C &2D). At 100 μg/ml, most of the cells go through apoptosis; and the increase of the apoptotic corps was noted (Figure 2E). The results indicated that DP extract induces the fragmentation of DNA of death HL-60 cells.

### Reactive specific oxygen (ROS) production by treated HL-60 cells with DP extract

**Figure 3:**
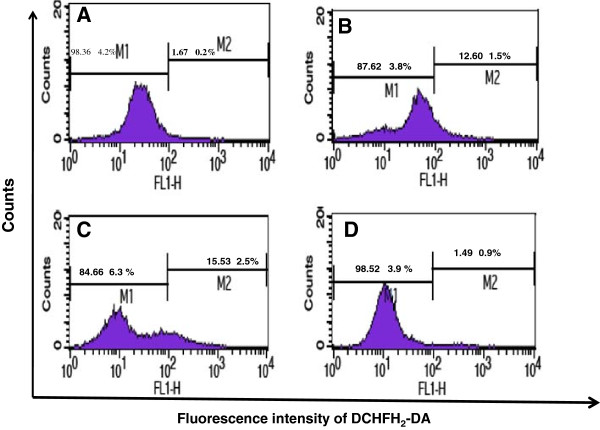
**Effects of extract DP on ROS production on HL-60 cells; Cells were treated with extract for 24 h followed by staining with DCHFH**_**2**_**-DA (5 μM), incubated for 30 min and the fluorescence in the cells was immediately analyzed using flow cytometry.** Data are presented the fluorescence intensity: **(A)**: control; **(B)**: 20 μg/ml; **(C)**: 50 μg/ml; **(D)**: 100 μg/ml.

ROS are generated in and around mitochondria and are regarded as the byproducts of normal cellular oxidative processes. As many anticancer drugs and DNA damage-causing agents activate the apoptotic pathway through ROS generation, the possibility that ROS elevation is a key step in DP-induced apoptosis was assessed using DCFH_2_-DA. The results showed that the intracellular ROS production of treated HL-60 cells increased 7.54 and 9.32 folds respectively at 20 and 50 μg/ml compare to the control (Figure 3). However the ROS production at 100 μg/ml was almost equal to the control the death of cells.

### Effect of DP extract on the mitochondrial membrane potential of HL-60 cells

Mitochondria play important role in the propagation of apoptosis and they are responsible for 90% of the energy needed for cells function. The disruption of mitochondrial integrity is one of the early events leading to apoptosis. To assess whether DP extract affects the function of mitochondria, the potential changes in mitochondrial membrane were analyzed by employing Rodamine-123, a dye which indicates the loss of mitochondrial membrane potential.

**Figure 4:**
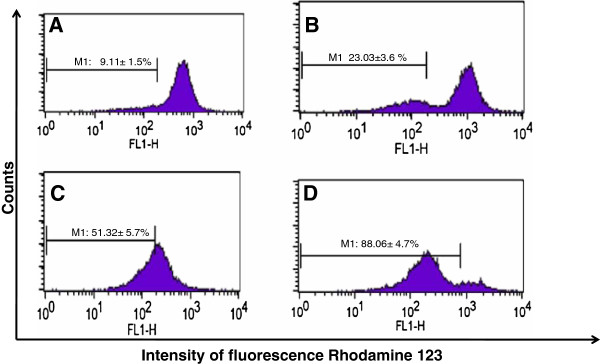
**Effects of DP on the integrity of mitochondrial membrane of HL-60 cells after 24 h.** Data are presented the fluorescence intensity: **(A)**: control; **(B)**: 20 μg/ml; **(C)**: 50 μg/ml; **(D)**: 100 μg/ml.

The results showed that the untreated HL-60 cells retained 90.89% of fluorescence. After 24 h of treatment cells with DP extract, the decline of fluorescence was enhanced in a concentration dependent manner from 23.03% at 20 μg/ml to 88.13% at 100 μg/ml (Figure 4). This result confirmed that DP extract induced apoptosis through the disruption of mitochondria membrane potential. This finding supports the view that alteration of mitochondria functions play a major role in the apoptosis in particular in cell death induced by chemotherapy [21].

### Effect of DP extract on DNA content and cell cycle of HL-60 cells

**Figure 5:**
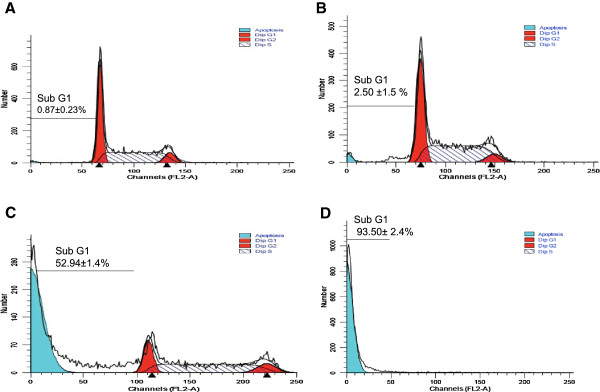
**Cell cycle analysis of DP extract on HL-60 cells after 24 h. (A)**: Control; **(B)**: 20 μg/ml; **(C)**: 50 μg/ml; **(D)**: 100 μg/ml. Data shown are expressed mean ± SD, Values affected with different letter are significantly different in the group, (p < 0.05).

The flow cytometric analysis of propiduim Iodide (PI)-stained was used to investigate the effect of DP extract on cell cycle progression on HL-60 cells. The results showed that the normal distribution cell cycle in the control. After treatment with extract at different concentration, the accumulation of cells was found in apoptotic phase (subG1) with significant modification of G2/M and S phases at the concentration of 20 and 50 μg/ml (Figure 5). Cells treated with 100 μg/ml of extract resulted to extensive accumulation of subG1 phase and loss of normal profile of the cell cycle.

**Figure 6:**
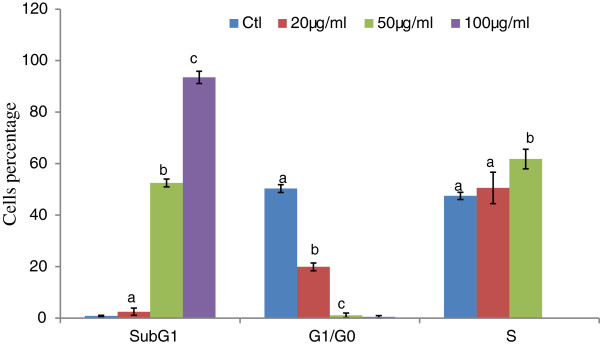
**Effect of DP extract on cell distribution after 24 h.** Data represents mean ± S.D. of three experiments. *p* < 0.001 was considered significantly different in comparison with the control.

The results demonstrated that DP extract induced apoptosis on HL-60 cells through cell cycle arrest. The proportion of cells in subG1 and S phases increased whereas cells in G1/Go phase decreased significantly in a concentration dependent manner compare to the control (Figure 6). The level of variation is between 2.87 - 107.43 and 2.52 -100.68 fold respectively for subG1 and G1/Go.

## Discussion

Recently, important attention has been focused on identifying natural substances capable of inhibiting or retarding the process of different stages of carcinogenesis. Anticancer drugs from natural sources having minimum side effects, inducing apoptosis, and targeting specific cytotoxicity to the cancer cells are the drugs of choice [22]. Finding novel natural compounds with low toxicity and high selectivity of killing cancer cells is an important area in cancer research. Anticancer drugs act by interfering with cell proliferation or, in most cases, by inducing programmed cell death, known as apoptosis [23]. Our studies revealed that DP extract demonstrated cytotoxicity activity on HL-60 and PC-3 cell lines with IC_50_ of 12 and 18 μg/ml respectively. These values are lower than 20 μg/ml (Figure 1). According to the US NCI plant screening program, a crude extract is considered to have *in vitro* cytotoxic activity, if the IC_50_ value following incubation between 48 and 72 h is less than 20 μg/ml [24]. Previous studies have indicated that root extract of *DP* has inhibitory activity against various cancer cell lines [12]. Cytotoxicity of DP extract arises its ability to interact with proteins, DNA via several functional groups by ionic interaction [25] or by DNA intercalation [26]. Literatures data on the cytotoxicity and apoptosis properties of DP extract are scarce. Our study is the baseline study on cytotoxicity and the apoptosis inducing properties of DP extracts on HL-60 cells. Apoptosis provides a number of clues with respect to effective anticancer therapy, and many chemotherapeutic agents reportedly to exert their antitumor effects by inducing apoptosis in cancer cells. Three apoptosis parameters of the intrinsic mediated apoptosis pathway have been investigated in our study with the HL-60 cells (i) apoptosis mediated by cell cycle arrest through the fixation to the receptors ; (ii) apoptosis mediated by mitochondria-involved signaling; (iii) the reactive oxygen species (ROS) induced apoptosis.

Cell cycle arrest is one of main the targets of many anticancer drugs such as camptothecin, doxorubicin, cisplatin, 5-fluorouracil. It has been shown that the ability of molecules/drugs to arrest cell cycle in G2/M or S phase was related to their sensitivity and increased with cell resistance [27]. Our results showed the increase of apoptotic cells, G2/M and M phase in a concentration dependent manner when the concentration of extract was raised compare with the control (Figure 6). This result showed that extract could act at all the stages of HL-60 cell cycle in a concentration-dependent and can be ranged among the cell cycle with non-specific agents. Several studies have reported that apoptosis involves a disruption of mitochondrial membrane integrity is decisive for the cell death process [28-30] and the depolarization of mitochondrial membrane potential is a characteristic feature of apoptosis. The evaluation of the effects of DP extract on the mitochondrial membrane potential (∆ψm) demonstrated the increase of loss of intensity of fluorescence respectively 2.52%; 5.62% and 9.66% fold at 20, 50 and 100 μg/ml. The decline of the fluorescence confirmed the death of the treated-HL-60 cells through the depolarization of their mitochondrial membrane potential. The finding confirmed that DP extract induces apoptosis of HL-60 through the disruption of mitochondrial membrane potential. This result supports the concepts that mitochondria are one of the most important organelles in cells which play critical roles in the mitochondrial apoptosis signal transduction pathway [22]. The loss of membrane potential is an early event in mitochondrial-mediated apoptosis [31]. After the reduction of membrane potential and the release of mitochondrial cytochrome C, a critical step is the formation of apoptosomes.

Identification of some features characteristic of apoptotic morphology such as degradation of DNA and apoptotic bodies (Figures 2D &2E) confirm the specific cleavage of DNA at sites between nucleosomes and the formation of a “ladder’ an indicator of apoptosis by DP extract [32]. The relationships between the mitochondrial dysfunctions observed during aging ROS production is still debated [33]. A disruption of mitochondrial membrane potential was observed in HL-60 cells exposed to oxidative stress provoked by duocarmycin A [34] and the elevation of the cellular ROS production can promote apoptosis [35]. Over production of ROS results in oxidative damage including lipid peroxidation, protein oxidation and DNA damage. A number of studies have shown that the phytochemicals involved in ROS production can selectively kill the cancer cells by raising the level of ROS above a toxic threshold [30,36,37]. HL-60 treated cells increase ROS production for almost 7.54 and 9.33 fold at 20 and 50 μg/ml respectively. These results confirmed that ROS were crucial in the induction of apoptosis and acted as upstream signaling molecules to initiate cell death. Besides apoptosis, the reduction in cell viability was further due to an arrest in the G2/M phase, the concentration dependent decline of mitochondrial membrane potential and increased of ROS production on HL-60 cells.

Identification of the phytochemicals compounds responsible of apoptosis may have important on anticarcinogenic activities [38]. Studies have been reported the presence of several bioactives compounds in DP extracts such as flavonoids, furano-coumarins, phenylated flavonoids [16,17]. Several studies demonstrated the mechanistic induction of apoptosis of different main groups of compounds present in DP extracts such as phenylated flavonoids [39,40] benzofuran [41]. Reports showed that plant-derived phenylated flavonoids and benzofuran, are cytostatic against various human cancer cell lines and induces apoptosis [42]. Therefore, our data suggested the possibility that DP might penetrate into cells and directly target mitochondria to increase membrane permeability and decrease (∆ψm) accompanied by ROS production.

## Conclusion

The present study indicates that DP extract can effectively inhibit proliferation of HL-60 and PC-3 cells. The apoptosis induction of HL-60 cells is accompanied by the production of ROS, the perturbation of mitochondrial membrane function.

## Abbreviations

MTT: 3-(4,5-dimethylthiazole-2-yl)-2,5-diphenyltetrazolium bromide; PI: Propidium iodide; Rh-123: Rhodamine- 123; DCFH2-DA: 2’, 7’-dichlorodihydrofluorescin diacetate.

## Competing interests

The authors declare that they have no competing interests.

## Authors’ contribution

PCA. Design, perform the study and write the manuscript; SG and SK help to perform the study; AP and NB collected the plant and carried out the extraction; SB and NYJ provided technical support and correct the manuscript, AKS provided all the reagent and chemical. All authors read and approved the final manuscript.

## Pre-publication history

The pre-publication history for this paper can be accessed here:

http://www.biomedcentral.com/1472-6882/13/223/prepub
